# Evaluation of Work-Related Personal Exposure to Aerosol Particles

**DOI:** 10.3390/toxics10070405

**Published:** 2022-07-21

**Authors:** Lina Davulienė, Abdullah Khan, Sergej Šemčuk, Agnė Minderytė, Mehri Davtalab, Kamilė Kandrotaitė, Vadimas Dudoitis, Ieva Uogintė, Martynas Skapas, Steigvilė Byčenkienė

**Affiliations:** 1 SRI Centre for Physical Sciences and Technology, Savanorių Ave. 231, LT-02300 Vilnius, Lithuania; abdullah.khan@ftmc.lt (A.K.); sergej.semcuk@ftmc.lt (S.Š.); agne.minderyte@ftmc.lt (A.M.); mehri.davtalab@ftmc.lt (M.D.); kamile.kandrotaite@ftmc.lt (K.K.); vadimas.dudoitis@ftmc.lt (V.D.); ieva.uoginte@ftmc.lt (I.U.); martynas.skapas@ftmc.lt (M.S.); steigvile.bycenkiene@ftmc.lt (S.B.); 2 Faculty of Physics, Vilnius University, Universiteto Str. 3, LT-01513 Vilnius, Lithuania

**Keywords:** aerosol particles, traffic emissions, PM exposure, deposition dose

## Abstract

The effects of air pollution on the general public received much attention recently. Personal exposure and deposition fraction of aerosol particles were studied in Vilnius, Lithuania, focusing on individuals working in an office and driving to work. Aerosol monitoring in the urban background was found to give an indication of the minimum concentrations of particulate matter (PM) expected at urban roads, as these correspond to the lowest PM concentrations measured there. In March 2021, PM_2.5_ concentrations at the urban background monitoring station reached values above the annual limit of 5 μg/m^3^ the World Health Organization in 50% of cases. Our study shows significant differences in exposure to air pollution in a car cabin and in a modern office. According to the multiple-path particle dosimetry model, the exposure of the person in the office is about 14 times lower than driving a car, where the minute deposition dose for PM_1_ is 0.072 µg/min for the period when the PM_2.5_ concentration in the urban background reaches 10 µg/m³. Compared to the PM_2.5_ mass concentration at the urban background station, the mean PM_2.5_ concentration in the vehicle reaches values that are 2–3 times higher. During the working day, when driving takes less than 10% of the time considered (commuting plus working), PM exposure during driving accounts for about 80% of the PM exposure caused by PM concentration in the office.

## 1. Introduction

Air pollution is a significant cause of mortality and diseases, and is estimated to be responsible for more than 6.7 million deaths worldwide in 2019 [1]. After years of research on the health effects of particulate matter, in 2000 the World Health Organization (WHO) first published the relative risk estimates for the effects of long-term particulate matter exposure on morbidity and mortality associated with a 10 µg/m^3^ increase in PM_2.5_ or PM_10_ concentrations as air quality guidelines (AQG) [2]. In 2005, the WHO published guideline values for the annual mean PM_2.5_ or PM_10_ concentration of 10 and 20 µg/m^3^, accordingly [2]. In 2021, new updated air quality guidelines were published, where much lower annual mean PM_2.5_ concentrations were provided, and it was recommended to not exceed 5 µg/m^3^, while 24 h average exposure should not exceed 15 µg/m^3^. The corresponding PM_10_ guideline values were calculated from the PM_2.5_ values, multiplied by a factor of three (for the previous AQGs the factor was two). Despite growing awareness and concern, the proportion of the global population exposed to the annual mean PM_2.5_ concentration of 10 µg/m^3^ reached almost 92% in 2019. Globally, the current level of air pollution has already become a major problem, and is estimated to shorten life expectancy by 1.8–2.9 years [1,3]. According to the EEA report “Air quality in Europe 2021”, exposure to fine particulate matter caused 307,000 premature deaths in 2019 [4]. Despite emission reductions, 97% of the urban population in the European Union was exposed to particulate matter levels above the latest WHO guideline values, and 71% of the urban population was exposed to a PM_10_ annual WHO limit value (15 μg/m^3^) in 2020 [4].

No limit values for the PM_1_ have been set by the WHO yet. The particles in the submicron size range have a low clearance rate in the alveolar region, which is the most sensitive area of the lung [5]. The deposition of ultrafine particles in this region raises the possibility of these particles entering the bloodstream and being transported to the heart, which can lead to severe biological cell damage, cardiac dysfunction, such as cardiac arrest, and oxidative stress [6]. In addition, ultrafine particles are associated with upper and lower respiratory tract damage and retardant lung growth [7], thus, starting malignant growth and causing cancer of the lungs [8].

In 2022, PM emissions from road traffic were one of the main emission sources in Europe. Compared to emissions from biomass burning for residential heating, emissions from transport significantly affect air quality in urban areas [9]. Epidemiological studies suggest that traffic emissions have an adverse effect on health [10], particularly among commuters (drivers and car passengers) and pedestrians [11]. Commuting is one of the main reasons for daily trips. According to the statistical data for the EU, commuting accounts for between 27 and 47% of the total distance travelled in the EU (Eurostat, passenger mobility statistics, 2021). The most common mode of transport in the EU is the car (56%), with the share ranging from 30 to 90% depending on the country [12]. In terms of direct transport emissions, it should be noted that road transport pollution is often higher than in other urban areas [13,14]. People only spend about 4–7% of their time daily in a transport microenvironment, although urban transportation accounts for a significant proportion of commuters’ daily integrated PM_2.5_ exposure (up to 12%).

Indoor air quality was studied extensively in recent decades because, in addition to outdoor sources, a variety of indoor sources play a significant role in daily pollution exposure [15]. During a typical workday, workers spend one-third of the day in the office. Modern office buildings are equipped with recuperative systems and some type of filtration. The operation of mechanical ventilation can reduce the infiltration of PM_2.5_ into indoor air, although this depends on the filtration system used [16]. In the FTMC office building equipped with the F1–F9 filtration system, the capture rate decreases with decreasing particle size, resulting in an average I/O ratio of 0.72 for particles with an aerodynamic diameter of 0.5 µm.

Knowledge of the concentrations to which individuals are exposed is critical for estimating the disease burden associated with particulate matter exposure. The health effects of the inhaled aerosol particles depend on the number of particles deposited and the region of deposition in the respiratory tract. Particle exposure and deposition in the lungs can be expressed using a variety of metrics. In addition to calculating lung deposition based on particle number or mass, it is also important to consider the deposition based on surface area, because particles interact directly with lung tissue through their surface [17,18].

The Environmental Protection Agency (EPA) maintains a sparse network of local air quality monitoring stations located in four fixed sites in Vilnius. Air quality modelling shows that air pollutant concentrations, PM_10_ in this particular case, tend to exceed the established limit value in densely populated areas, i.e., in the city centre and in the outskirts. The daily PM_10_ concentrations in the urban environment show a strong gradient over distances as short as 10 m, which is particularly evident along major roads [19].

This study is a case study aimed at determining the exposure of an individual to PM mass concentrations during the working day in Vilnius, Lithuania, during the period when the PM_1_ urban background concentration is above the WHO limit values. The total deposition dose and particulate deposition fraction in different lung regions were evaluated. The analysed period of PM exposure included travel time by private vehicle and work time in the office, while particulate matter concentrations were measured both inside and outside the car and the office building. Another purpose of this work was to show that low-cost environmental monitoring sensors can be integrated into the MPPD model. Some model limitations were overcome by mathematical transformation of sensor data.

## 2. Materials and Methods

### 2.1. Location and Measurement Setup

**Figure 1:**
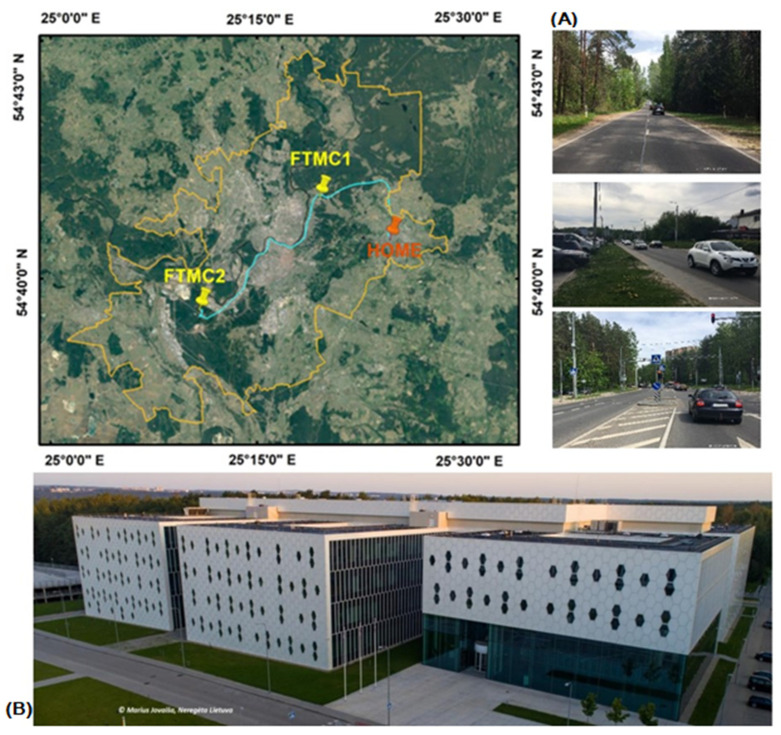
Mobile monitoring routes with points representing the place of residence (red pin) and the workplace (FTMC1 and FTMC2, yellow pins) (**A**); street (**B**) and office (FTMC1). The fixed measurement location (urban background) coincides with the location of FTMC1.

Personal exposure to PM during daily commuting between home and office (later referred to as sites FTMC1 and FTMC2) was assessed in the city of Vilnius (54°41′13.2″ N, 25°16′44.4″ E). Vilnius is the capital of Lithuania, with a population of ~600,000 and a population density of 1372 inhabitants/km^2^ in 2022. The climate of the city is classified as moderately continental, with four distinct seasons—warm summer, cold winter, transitional spring, and autumn. Driving routes were recorded on 17 and 18 March 2022, between 7:00 and 20:00 local time (LT), with a selection of points representing the employee’s daily route through different environments, such as urban background areas, residential areas, industrial areas, high-traffic areas, and commercial areas with high air pollution levels (Figure 1).

The location of the fixed site used for aerosol particle number, mass concentration, and size distribution measurements has no direct contribution from multiple pollutant emission sources and was used to represent urban background pollution. The site is located 6–7 km northeast of the city centre, and is shielded by a forested area from a busy road (8400 vehicles per day) and a smaller road (6200 vehicles per day) (Figure 1) [20]. The measuring equipment of the reference monitoring station was placed on the roof of the academic building of the SRI Centre for Physical Sciences and Technology (FTMC, www.ftmc.lt, accessed on 5 July 2022).

Indoor air quality measurements were performed in a laboratory space that could also be described as a typical office with a three-stage filtration air supply system (for a more detailed description, see [21]).

### 2.2. Methods and Measurements

#### 2.2.1. Field Campaign

The low-cost environmental monitoring sensor URAD Monitor A3, version 9, SC MAGNASCI SRL, Dumbravita, Romania [22], was used to measure the main air quality parameters while driving over the selected route. This monitor records a total of 10 parameters such as: air temperature, atmospheric pressure, air humidity, PM_1_, PM_2.5_, and PM_10_, etc. The concentration of PM in the air is determined by a high-quality laser scatter sensor. From the comparative study of the URAD monitor A3 with quartz filter measurements according to SR EN 12341:2014 [23], it appears that the monitor measures with an uncertainty of ±12% from the limit value (LV_2.5_ = 35 μg/m^3^) in the case of PM_2.5,_ and ±11% from the limit value (LV_10_ = 50 μg/m^3^) in the case of PM_10_. This statement is valid for concentrations >~10 μg/m^3^ in both cases.

To display the spatial distribution of PM concentration on the map, the results obtained on each route were smoothed and converted to shapefiles (point vectors) using ArcGIS version 10.6 software, ESRI, Redlands, CA, USA, 2018. For each sampling campaign, interpolation was performed using the inverse distance weighting (IDW) method.

#### 2.2.2. Fixed-Site and Indoor Measurements

The fixed site and indoor measurements in the office room were performed at the FTMC1 site. Measurements of aerosol particles in the size range of 0.54 to 20 µm were made using the aerodynamic particle sizer (APS, TSI model 3321). The type of aerosol inlet was defined as a hood. The main inlet was located 16 m above the ground and was elevated 2 m above the roof. The sampling line was located inside the manifold, and the flow rate was 5 L/min. No impactor was used in the sampling line leading to the APS. The APS measurement lasted 4:50 min, and was repeated every 5 min. It was estimated that the APS had better detection accuracy for solid particles than for liquid particles [24]. The counting efficiencies in the size range of 0.8–10 μm varied between 85 and 99% for solid particles, and between 75 and 25% for liquid particles.

Due to the many unknowns in the study area, the mass conversion parameters were simplified by assuming a particle density of 1.0 g/cm^3^ and a spherical shape of the particles, i.e., the aerodynamic shape parameter was equal to 1. These fixed parameters were the same regardless of the environment and the study scenario.

### 2.3. Multiple-Path Particle Dosimetry Model

The multiple path particle dosimetry (MPPD) model, version 3.04, was provided by Applied Research Associates Inc., Raleigh, NC, USA, 2016. The MPPD model was used as a computational tool to calculate the deposition of polydisperse aerosols in the upper respiratory tract (URT), tracheobronchial region (TB), and alveolar region of an adult human for particle sizes from ultrafine (1 nm) to coarse (10 µm). The aerosol particle size range of 0.5 to 10 µm was covered by APS, and used to calculate the input parameter, i.e., GSD, in the MPPD model. The model was run for multiple path techniques to monitor airflow and to calculate aerosol deposition in the lungs during home–office travel. The deposition in each airway region was calculated using theoretically derived efficiencies for deposition by diffusion, sedimentation, and impaction in the airways or at airway branches. Filtration of aerosols through the nostrils is calculated using empirically derived efficiency equations. To run the MPPD model, the input parameters, such as the median mass diameter (MMD), geometric standard deviation (GSD), and PM values, were estimated from field measurements (Table S1). The PM_x_ concentration values were taken from the URAD monitor A3 measurements. GSD values were derived from the APS mass estimation data and are presented in Table S1. Measurements made with low-cost environmental monitoring sensors usually lack such details as MMD, GSD, etc. The MMD values used in this study were assumed to be 10, 2.5, and 1 μm for PM_10_, PM_2.5_, and PM_1_, respectively. By using the fractions of PM_1__–__2.5_ and PM_2.5__–__10_ data, instead of PM_2.5_ and PM_10_ as inputs to the MPPD model, we were able to reduce the influence of PM_1_ for particles >1.0 μm, and the influence of PM_2.5_ for particles >2.5 μm. In the case of PM_2.5_ and PM_10_, the input parameters for fractions PM_1__–__2.5_ and PM_2.5__–__10_ were recalculated, and the final result was balanced accordingly:

f(PM_2.5_) = (f(PM_1_) × PM_1_ + f(PM_1__–__2.5_) × PM_1__–__2.5_)/PM_2.5_ (1)

f(PM_10_) = (f(PM_2.5_) × PM_2.5_ + f(PM_2.5__–__10_) × PM_2.5__–__10_)/PM_10_ (2)

Since the office worker spends most of the workday indoors, e.g., in the building or in the vehicle, only indoor environment settings were used in the MPPD model. The breathing frequency was used 20 L/min. The following assumptions for stimulating deposition in the airways of an adult human, such as a functional residual capacity volume of 3300 mL, a tidal volume of 625 mL, an inspiratory fraction of 0.5, and a particle density of 1 g/cm^3^ are given by the International Commission on Radiological Protection (ICRP) and model recommendations.

### 2.4. PM Deposition Dose

A general model for assessing the deposition dose DD of aerosol particle in the human respiratory tract, as summarised in [25], is based on measurements of aerosol mass concentration in air in different size ranges, and breathing parameters such as airflow rate in lungs or ventilation, which might differ according to the focus group (gender, age, activity level, etc.):

DD = DF × MV × Σ_i_(PM_i_ × Δt_i_) (3)

where MV is the minute ventilation or airflow in the lung (m^3^/min), PM_i_ is the particulate matter concentration during time step i, and Δt_i_ is the duration of time step i (min). The deposition fraction DF may be experimentally evaluated [25] or calculated, e.g., based on the method approved by the International Commission on Radiological Protection [26]. In this paper, the MDDP model was applied to evaluate the DF during different time periods when the field experiments were carried out. The minute deposition dose MDD was calculated, taking into account the time of exposure (in minutes) to allow comparison between different exposure episodes [7]. The average PM mass concentration values from the field experiment that were used as input data in the MPPD model for deposition calculation during all the trips are presented in Table 1. The estimation of total size-segregated PM deposition fraction throughout the entire human airways is important for further research of the PM regional deposition.

### 2.5. Transmission Electron Microscopy Analysis

Indoor and outdoor filter samples were imaged digitally using transmission electron microscopy (TEM). Images and EDX spectra were acquired using a Tecnai G2 F20 X-TWIN (FEI, The Netherlands, 2011) microscope with a Schottky-type field emission electron source, a high-angle annular dark field (HAADF) detector, a single- and double-tilted sample holder, and an 11 MPix ORIUS SC1000B (Gatan) CCD camera. Samples were mounted directly on a holey carbon-coated copper grid (Agar Scientific Ltd., Stansted, UK).

## 3. Results

### 3.1. Overview of Background Measurements during Study

**Figure 2:**
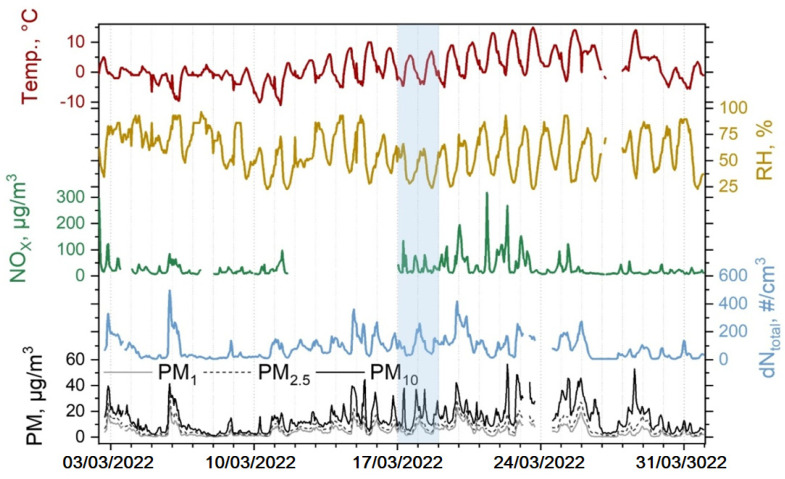
Fixed measurements of meteorological parameters: temperature (red), relative humidity (yellow), NO_x_ concentration (green), total particle number concentration N_total_ (blue), and PM_1_, PM_2.5_, PM_10_ mass concentrations (black, dotted line, grey, respectively) during March 2022 (experiment days are marked in blue rectangle).

The average daily temperature in March 2022 was 1.4 °C and the relative humidity (RH) was 59.7%. On 17 and 18 March, the highest air temperature values of 5.5 to 7.0 °C were reached at noon (2–5 p.m.), and the lowest values of −4.5 to −4.0 °C were reached in the early morning (6–7 a.m.). On these days, RH varied from 24 to 66% (Figure 2). The average daily NO_x_ concentrations are 33.2 µg/m^3^ and 32.1 µg/m^3^ during the field test, while March monthly average is 33.3 µg/m^3^. NO_x_ concentration shows that no pollution events (e.g., wildfires) occurred during the days of the field experiment on 17 and 18 March. The concentration peaks of NO_x_ correlate well with PM_1_, PM_2.5,_ and PM_10_ concentrations (0.72, 0.74, and 0.7, respectively) during the measurement campaign.

The maximum daily average particle number concentration during the experiment in the size range of 0.5–20 µm is 260 #/cm^3^, while the lowest daily average is 30 #/cm^3^. The aerosol particle number concentration varies between 5 and 500 #/cm^3^ during March, with a monthly mean total number concentration N_total_ (standard deviation) of 85 (80) #/cm^3^.

**Figure 3:**
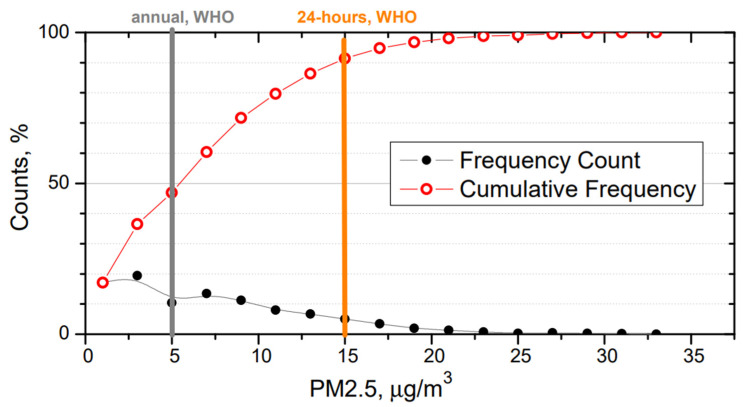
Frequency count and cumulative frequency of PM_2.5_ in March 2022 measured at the urban background station in Vilnius along with WHO annual (bold grey line) and 24 h (bold orange line) limits for PM_2.5_.

The mean PM concentrations during March are 4.7 (4.2), 7.5 (5.6), and 14.5 (10.4) μg/m^3^ for PM_1_, PM_2.5,_ and PM_10_, respectively. During the experiment days, the mean daily PM_1_, PM_2.5,_ and PM_10_ concentrations are found to be 5.2 (2.9), 7.8 (4.0), and 15.8 (8.9) μg/m^3^, respectively. These results suggest that field experiment was conducted during days with a regular atmospheric condition, and no extreme pollution event had any effect on the field experiment. PM_2.5_ mass concentration in the urban background reaches levels that are higher than the WHO annual limit of 5 μg/m^3^ during ~50% of measurement campaign (Figure 3). The 24 h limit (15 μg/m^3^) set by the WHO is exceeded during ~10% of measurement campaign.

**Figure 4:**
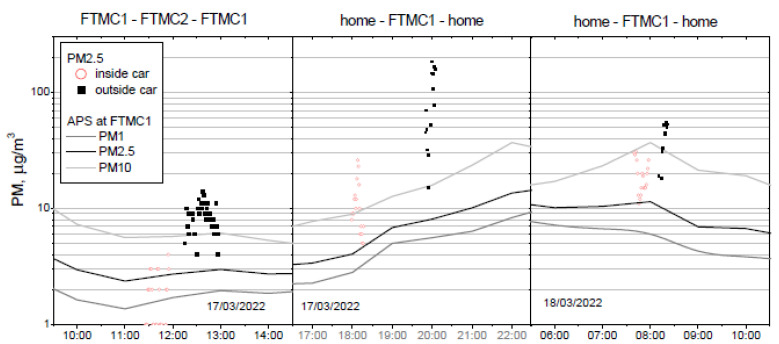
Timing of field experiments and PM_2.5_ mass concentrations measured during each route inside (red circle) and outside (black rectangle) the vehicle, and mass concentration of PM_1_ (grey line), PM_2.5_ (black line), and PM_10_ (light grey line) at the urban background site located at FTMC1.

The three field experiments were performed at different times of the day, and at two different routes, to evaluate PM mass concentration inside and outside the vehicle. The first trip was made between FTMC main office (FTMC1) and laboratories (FTMC2), followed by two trips between home and FTMC1. PM_1_, PM_2.5,_ and PM_10_ mass concentrations were measured inside the car during the forward routes, and outside the car during the trips back (Figure 4). The mean urban background PM mass concentrations during the mobile monitoring period are shown in Table 2. The highest PM mass concentration is observed in the morning (PM_10_ = 36.9 µg/m^3^) due to rush hour, while the lowest PM mass concentration (PM_10_ = 5.7 µg/m^3^) is determined during the midday route (FTMC1–FTMC2–FTMC1).

For all the periods of the field experiment, the urban background level of PM_2.5__–__10_ has the highest contribution (47 to 69%), followed by PM_1_ (18 to 39%). Regarding PM mass ratios (Table 1), PM_1_/PM_2.5_ ratios are found to range from 57.0 to 73.5%, with the highest value of 73.5 for the evening route (FTMC1–home). In addition, PM_1_/PM_10_ and PM_2.5_/PM_10_ mass ratios range from 17.6 to 39.4%, and from 30.9 to 53.5%, respectively. It clearly shows that fine particles <2.5 µm account for up to 50% of the PM_10_ mass, except in the FTMC1–home route (30.9%). In general, fine particles (nucleation and Aitken mode particles) dominate in the urban environment due to the abundance of primary emission sources, but in the spring, the influence of winter road maintenance (sanding) could be important as the samples have a higher Si content.

Particle size distribution analysis (Figure S1) shows that the lowest number concentration (30 ± 3 #/cm^3^) of aerosol particles is observed during the FTMC1–FTMC2–FTMC1 route period, which is consistent with the observed lowest mass concentration. It is seen from Figure S1, that the aerosol number concentration in the urban background during this route is almost three times lower than the integrated mean value during 17–18 March (100 ± 10 #/cm^3^). The highest integrated aerosol particle number concentration (140 ± 10 #/cm^3^) is observed during the home–FTMC1–home (morning) route, ranging from 90 #/cm^3^ (0.5 µm) to 1 × 10^−5^ #/cm^3^ (20.0 µm). In addition, the values of integrated number concentration on the route home–FTMC1–home (evening) are similar to the mean values for the whole month of March and for mobile monitoring days: 100 ± 10 #/cm^3^, 80 ± 10 #/cm^3^, 100 ± 10 #/cm^3^.

### 3.2. Overview of Mobile Monitoring Results

**Figure 5:**
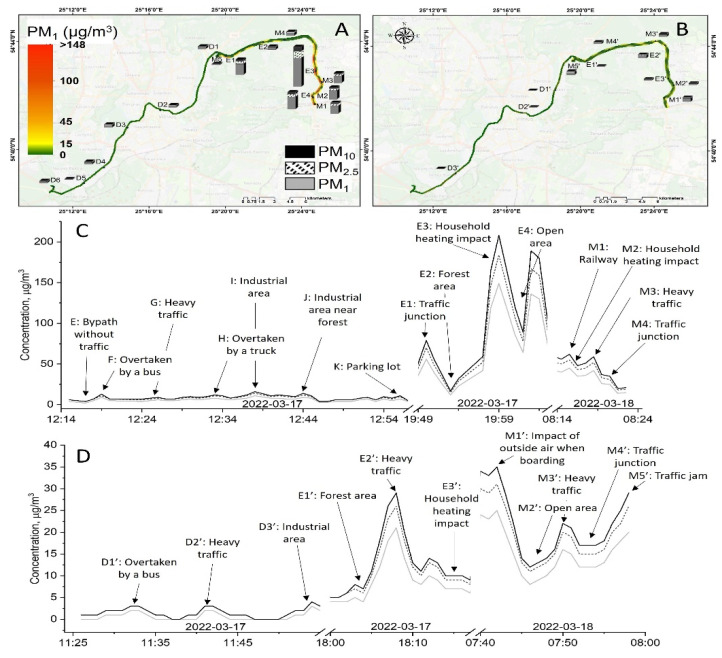
Spatial distribution of BC and PM concentrations for the route home–FTMC1–FTMC2 outside the car (**A**) and inside the car (**B**). Coloured line in the map marks PM_1_ concentration, columns next to the route mark concentrations of PM_1_, PM_2.5_, PM_10_. Time series of PM concentrations outside the car (**C**) and inside the car (**D**).

Regarding the PMs mass concentration, it is found that mass concentrations during the mobile monitoring are much higher than those found in the urban background site (Figure 5) for most of the period.

The mean PM_2.5_ mass concentration inside the vehicle is generally about 2–3 times higher than outside (Figure 5). When background PM_2.5_ concentrations are stable, the relationship between the mean indoor and outdoor concentrations measured during the field campaign on the road shows the influence of the vehicle’s ventilation system. This is the case for the first (midday) and the third (morning) series of measurements. In contrast, due to the time lag between the evening series of measurements, background PM_2.5_ concentrations increases threfold between the series of measurements inside and outside the vehicle, resulting in the large difference (approximately nine-fold) between the in-vehicle and out-of-vehicle PM_2.5_ concentrations. Therefore, to compare these measurement series, the change in urban background should be taken into account.

The PM concentration measured in the urban background corresponds to the minimum values for PM_2.5_ measured both on the road and in the car, except for the measurements during the first trip in the car, where the PM concentration is lower than the urban background concentrations. In this campaign, the background levels are also the lowest, compared to other measurement campaigns (Figure 4). The lowest PM_2.5_ concentration on the road is measured in the forest area on the way from FTMC1 to the residential area outside the city (Figure 5).

PM_2.5_ concentrations measured on the road can exceed urban background levels many times over. For example, during evening driving, the PM_2.5_ concentration outside the vehicle increases up to 100 µg/m^3^ for about 3 min, and exceeds the background value by more than 10 times. At the same time, the PM_2.5_ concentration inside the vehicle is above 10 µg/m^3^ for about 6 min, and exceeds the background value for PM_2.5_ by a factor of five. The highest PM_2.5_ levels are measured on the road in the residential area with the most private houses. Compared to the PM mass concentration at the urban background station, the mean PM_2.5_ concentration at the road reaches values of 4 to 10 times higher, and inside the vehicle it reaches values of 2–3 times higher.

The PM_2.5_ mass concentration limits set by the WHO are as follows: 5 μg/m^3^ annual mean, 15 μg/m^3^ 24 h mean (WHO, 2021). The PM_2.5_ mass concentration exceeds the annual mean limit for all trips, except for the midday in-cabin measurements when the urban background concentration is low (Figure 4). In addition, the PM_2.5_ concentration in the in-cabin measurements also exceed the WHO limit for the 24 h average PM_2.5_ during the evening and morning trips (except for the evening in-cabin measurements).

**Figure 6:**
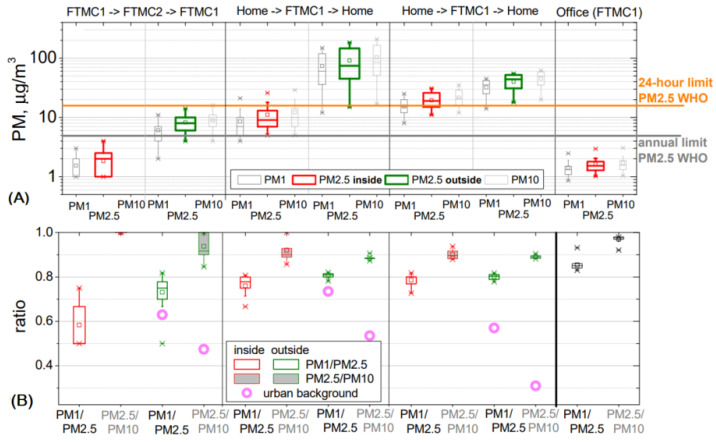
PM mass concentration and ratios of PM fractions: (**A**) statistics of mass concentration for all PM fractions inside (PM_2.5_ red) and outside (PM_2.5_ green) vehicle recorded while driving in Vilnius and in the office room; (**B**) ratios PM_1_/PM_2.5_ (empty box) and PM_2.5_/PM_10_ (filled light grey box). Whiskers: the 1st and 99th percentiles (error bars), quartiles Q_1_ and Q_3_ (box), median (horizontal line), and mean (square). PM ratios measured at the urban background station FTMC1 (APS) (purple circle).

According to the URAD measurements, the PM_1_ fraction dominates the total PM concentration during the field campaign, both outside and inside the vehicle (Figure 6). The ratios of PM_1_/PM_2.5_ and PM_2.5_/PM_10_ are about 0.80 and 0.9 for the evening and morning trips, respectively, indicating that the PM_2.5__–__10_ fraction accounts for only about 10% of the total PM concentration. Comparison of these ratios with the urban background PM_1_/PM_2.5_ and PM_2.5_/PM_10_ (purple rings, Figure 6) shows a different trend, as PM_2.5_/PM_10_ ratios are much lower compared to PM_1_/PM_2.5_, indicating that the PM_2.5_/PM_10_ fraction are large, or of comparable size to PM_1_. The contribution of PM_10_ particles varies between 50 and 70%. This difference between mobile and fixed measurements could be due to the fact that the mobile measurements were performed on the road, i.e., close to the sources of generation of the new particles. Another reason could be that the lack of isokinetic sampling inlet for URAD during the driving results in losses of larger particles in the PM_2.5_/PM_10_ fraction compared to the measurements at the urban background station.

The ratios PM_1_/PM_2.5_ and PM_2.5_/PM_10_ in a modern office are higher than obtained for other environments in the same period due to the peculiarities of the filtering system, in which the smallest particles are filtered less effectively [27].

### 3.3. Total and Regional PM Deposition Dose in Human Airways

**Figure 7:**
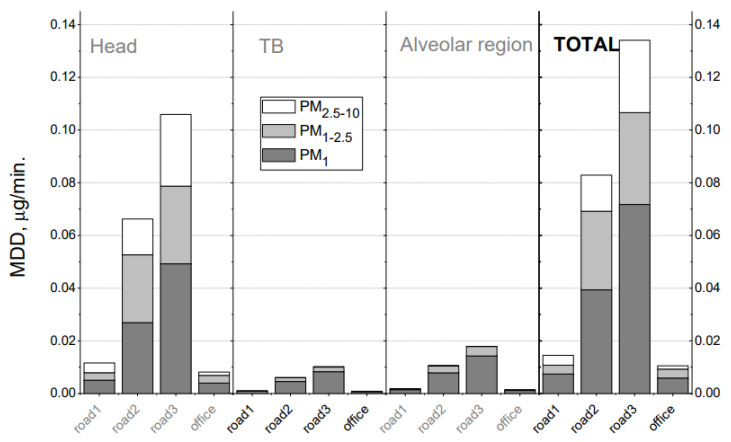
Contributions of PM fractions (PM_<1_, PM_1__–__2.5_, and PM_2.5__–__10_) to total deposition dose in URT, TB, and alveolar regions calculated with the MPPD model for different routes (“read1-3” corresponds to cases presented in Table 1) and for the modern office.

**Figure 8:**
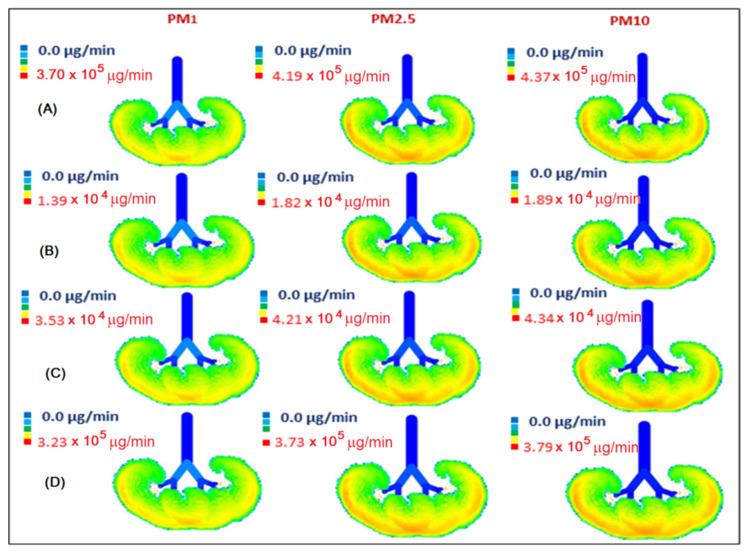
Visualization of size-segregated PM mass deposition rate in the human respiratory tract on the route FTMC1–FTMC2 during midday (**A**), from FTMC1–home during the evening (**B**), home–FTMC1 during morning rush hours (**C**), and in office FTMC1 (**D**).

The calculated inhaled deposited mass of PM during working hours in the office and during driving on all routes, as well as the minute deposition dose in the respiratory geometry, are shown in Figure 7 and Figure 8. According to the model, the applied DF depends on the aerodynamic size of particles, but not on the concentration of aerosols. Therefore, the DF values for PM in the office and in the car are the same for the same aerodynamic diameter, regardless of the difference in concentration. The total DF for PM fractions PM_<1_, PM_1__–__2.5_, PM_2.5__–__10_ are 38%, 90%, and 98%, respectively (Figure S2). The largest PM are predominantly deposited in the upper respiratory tract (head), while the finer PM fractions dominate in the lower airways. The total DF for PM_1_, PM_2.5_, and PM_10_ (calculated using formulas 1 and 2 shown above) is approximately 38%, 56%, and 65%, respectively, with a small standard deviation (3%) between the measurement series. The lowest MDDs are obtained for the midday commute when the person travels from FTMC1 to FTMC2 and the PM urban background concentrations are the lowest of the analysed period. In contrast to the rush hour, this could also be influenced by a combination of the height of the mixed layer during the day and the lower traffic intensity.

It is important to note that in our study we used normal respiratory parameters and the nasal route to estimate the deposition fraction, which may vary depending on the respiratory scenario. As shown in a study conducted in Hungary, the PM_1_ deposition fraction in the extra-thoracic region (head) decreases monotonically from 26% (for sleeping) to 9.4% (for heavy exercise) with a parallel increase in the alveolar region from 14.7% (for sleeping) to 34% (for heavy exercise) [28]. It is found that particle deposition differs significantly depending on the subject’s gender (male vs. female) and age (17–45% in adults vs. 10–23% in children; two times greater in adults) [29]. In our case, the PM1 value for a seated middle-aged man is found to be 26% in the head region and 8% in the alveolar region. It is lower in the alveolar region compared to the specimen study (14.7%), but this could be due to differences in particle size distribution. The deposition fraction of PM_1_ could also be underestimated in our results, since it was evaluated only for a diameter of 1 μm, i.e., not for a range.

Significant differences are found between indoor (office) and road deposition doses. Indoor PM concentrations are close to those found for drivers during midday at low urban background concentrations, and are about 8 (for PM_1_) to 14 (for PM_10_) times lower than deposition doses for drivers during rush hour. As a result of a better ventilation system ensuring air quality in FTMC1 (office buildings), a comparatively lower PM dose is deposited in the lungs of office workers than during on-road driving. Taking into account the time spent driving (40 min on average for driving from office to home) and in the office (8 h), the deposition doses (MDD multiplied by the time spent on a given activity) of total inhaled PM_1_ are in a comparable range: office/road = (0.006 × 8) / (0.072 + 0.039) × 1/3) ≈ 48/37 ≈ 1.3. This means that the fraction of daily PM_1_ exposure in the office may be higher than the fraction obtained on the road, depending on the time spent driving. During the workday, when driving takes less than 10% of the time considered (commuting plus working), PM_1_ exposure while driving accounts for about 80% of the PM_1_ exposure caused by office PM_1_ concentrations.

The MDD for the driver is about 14 times higher for PM_1_ and PM_10_ during the rush hour than in the office when the urban background concentration of PM_2.5_ is up to 10 µg/m^3^. During the low urban background concentration period, the in-vehicle PM concentration is lower than the background concentration by a factor of two. This may be due to the limitations of the mobile measurement device used.

The visualization of the size-segregated PM mass deposition rate in the human respiratory tract on the road and in the office (Figure 8) shows the large differences in MDD distribution within the portion of the respiratory tract shown. It is also important to note that MDD is dominated by the PM_1_ fraction, and exerts the greatest influence on the lowest part of the respiratory tract, the alveolar region.

**Figure 9:**
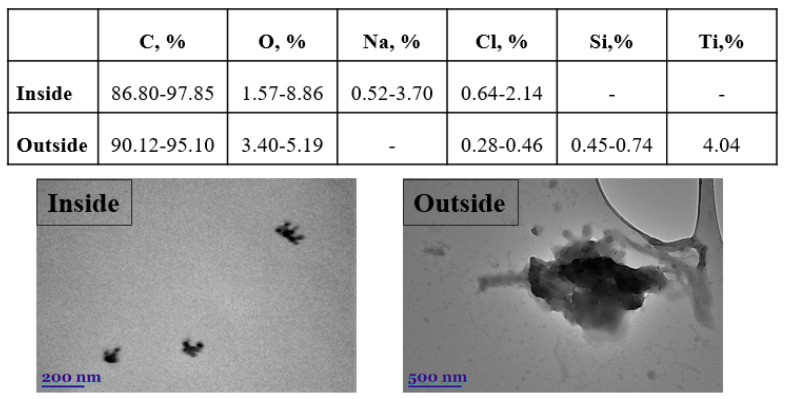
TEM images and EDX data of individual inside/outside particles.

The morphological characteristics of the particular matter (PM_1_ and PM_2.5_) were studied using transmission electron microscopy (TEM), while the elemental composition was determined using EDX spectra (Figure 9). The majority of the particles in the inside sample are aggregates of smaller, round-shaped nanoparticles. The smallest particles have a length of about 40 nm, and only a small number are longer than 200–300 nm. Compared with the inside, the outside sample contains more irregular particles larger than 1 µm. The smaller particles also form large ones with diameters of approximately 2.5 μm (i.e., PM_2.5_). Elemental analysis of particulate matter reveals the presence of mostly elemental/organic carbon and oxygen (Figure 9). Metals and inorganic salts are also discovered. The minor constituent elements sodium, chloride, silicon, and titanium contribute from 0.28% to 4.04%, to the total weight of the particles.

It is observed that the major constituent of the outside sample is silica (Si), which confirmed our above statement that, in addition to traffic emissions, the road dust is one of the major sources of exposure outside. Being inside a vehicle does not protect you from the emissions outside or small particles (Figure 9). Both PM_1_ and PM_2.5_ have the potential to cause significant cytotoxicity and genotoxicity in cells. The toxic effects of PM_1_ are stronger than those of PM_2.5_. PM_1_ contains more toxic and carcinogenic compounds than fine and coarse particles, and can penetrate deeper into the respiratory tract. The combined effects of size and chemical composition may be responsible for the stronger cytotoxic effects, whereas chemical species may be primarily responsible for the genotoxic effects of PM_1_ [30]. The most important protection for humans against outdoor air pollution in vehicles is ventilation, air conditioning, modern filters, and recirculation systems. Studies show that particle filters without activated carbon in cars reduce particulate matter by 46%, while using filters with activated carbon reduces it by 74%, compared to unfiltered air [31]. Transmission electron microscope analysis (TEM) confirms that the outdoor sample is dominated by a large number of particles with a wide size range (PM_1_, PM_2.5_, and PM_10_). In contrast, the sample measured inside the vehicle shows that filters remove larger particles, leaving almost only particles whose size falls within the range describing PM_1_.

## 4. Discussion

Urban air pollution varies widely both spatially and temporally, and commuter exposure can vary substantially depending on the daily distance travelled between home and work. PM_2.5_ concentrations measured on the road can exceed background levels by many times. Of the two analysed routes, the time of the day when drivers and passengers could be exposed to the maximum PM concentrations was the morning hours. Urban background PM_2.5_ concentrations ranges from 5 to 10 µg/m^3^ during the morning and evening commutes. PM_2.5_ concentrations at the road are above the 24 h limit value (WHO) during these measurement sessions. Compared to the PM mass concentration at the urban background station, the mean PM_2.5_ concentration at the road reaches values of 4 to 10 times higher, and inside the vehicle it reaches values 2–3 times higher. Office PM concentrations are below the urban background concentration and the WHO limits for PM_2.5_.

Due to its high toxicity, the smallest PM fraction, PM1, attracts the most attention and is an important subject of study. The PM1 dominates in both environments, i.e., in the on-road vehicle and in the modern office, but mass concentrations differ significantly due to differences in infiltration. The MDD for the driver is about 14 times higher for PM_1_ (and PM_10_) during the rush hour than in the office when the urban background concentration of PM_2.5_ is up to 10 µg/m^3^. During the low urban background concentration period, the in-vehicle PM concentration is underestimated, due to the limitations of the mobile measurement device used.

During the workday, when driving takes less than 10% of the time considered (commuting plus working), PM_1_ exposure during driving comprises about 80% of the PM_1_ exposure caused by PM_1_ concentration in the office.

This study was the first attempt to evaluate PM pollution on Vilnius roads and in the driver’s cabin, and to compare it with measurements of the urban background. The analysed measurement series are not very extensive, as some measurement series were discarded when the measurements of the whole set of instruments were not available. It is shown that low-cost environmental monitoring sensors can be used together with the MPPD model to assess PM deposition on the road, but with some limitations in the case of low urban background concentrations.

## Figures and Tables

**Table 1:** Mean-size-segregated PM mass concentration in car cabin during the trips in Vilnius used as model input.

Routes	Date and Time	PM_1_ µg/m^3^	PM_1__–__2.5_ µg/m^3^	PM_2.5__–__10_ µg/m^3^
FTMC1–FTMC2	17/03 midday	1.6	0.3	0.3
Home–FTMC1	17/03 evening	8.5	2.6	1.1
Home–FTMC1	18/03 morning	15.3	3.1	2.2
Office FTMC1	18/03 working hours	1.3	0.3	0.1

**Table 2:** PM mass and number concentrations station and PM ratios during field experiment at the urban background.

Date and Time	PM, µg/m^3^			PM Ratios			Total Number Conc., #/cm^3^
	PM_1_	PM_2.5_	PM_10_	PM_1_/ PM_2.5_	PM_1_/ PM_10_	PM_2.5_/ PM_10_	N
17/03/2022 12:00–13:00	1.7	2.7	5.7	0.63	0.30	0.47	34
17/03/2022 19:30–20:30	5.0	6.8	12.7	0.74	0.39	0.54	97
18/03/2022 8:00–8:30	6.5	11.4	36.9	0.57	0.18	0.31	136
18/03/2022 8:00–17:00	3.6	6.0	15.8	0.61	0.25	0.40	67

## Data Availability

Not applicable.

## Supplementary Materials

The following supporting information can be downloaded at: https://www.mdpi.com/article/10.3390/toxics10070405/s1. Table S1: Geometric standard deviation values for PM deposition in the MPPD model, Figure S1: Average particle number size distributions during the field experiments and the entire study, Figure S2: The total deposition fraction (DF) for particulate matter (PM) fractions PM_<1_, PM_1__–__2.5_, PM_2.5__–__10_.
