# Supplementary material for: Evaluation of Work-Related Personal Exposure to Aerosol Particles
Source: Toxics. 2022 Jul 21;10(7):405. doi: 10.3390/toxics10070405 (PMC9321620; doi:10.3390/toxics10070405)
Supplement: Supplementary file 1 [file toxics-10-00405-s001.zip › toxics-1774858-supplementary.pdf]

Supplementary Material:

Table S1. Geometric standard deviation values for particulate matter (PM) deposition in the multiple-path particle dosimetry model (MPPD).

		$PM_{2.5-10}$	$PM_{1-2.5}$	PM_1
**Routes**	Date and time		GSD	
**FTMC1-FTMC2**	17/03/2022 midday	1.5	1.44	1.21
**FTMC1-HOME**	17/03/2022 evening	1.48	1.51	1.21
**FTMC1-HOME***	18/03/2022 morning	1.46	1.44	1.22
**Office FTMC1**	18/03/2022	1.56	1.48	1.19

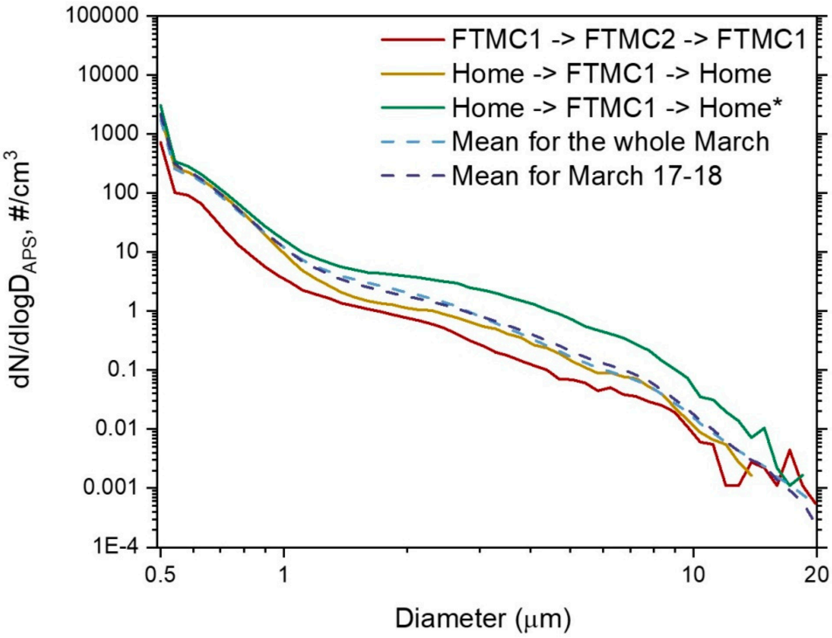

Figure S1. Average particle number size distributions during the field experiments and the entire study.

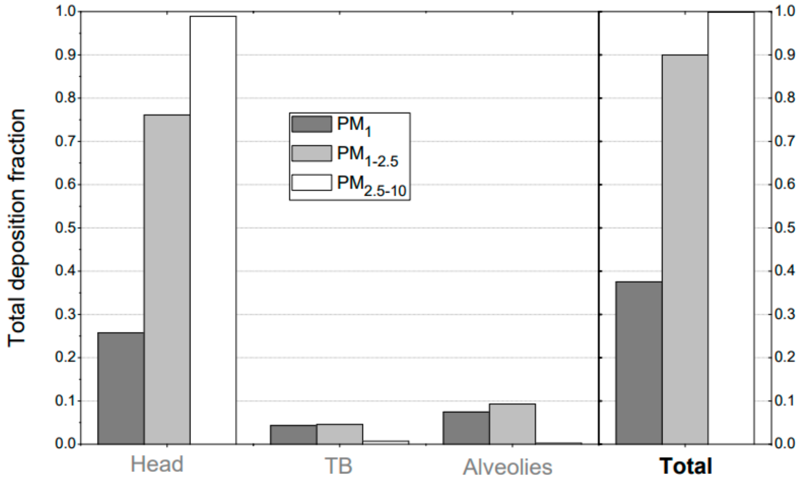

Figure S2. The total deposition fraction (DF) for PM fractions $PM_{<1}$, $PM_{1-2.5}$, $PM_{2.5-10}$
